# Impact of a smoking cessation program on smoking prevalence and food security among food pantry users – a study protocol for a pragmatic cluster randomised controlled trial

**DOI:** 10.1186/s12889-020-09232-0

**Published:** 2020-07-17

**Authors:** Anja Simmet, Michael Teut, Romy Schleicher, Andreas Bschaden, Nanette Stroebele-Benschop

**Affiliations:** 1 Institute of Nutritional Medicine, Department of Nutritional Psychology, University of Hohenheim, Fruwirthstr. 12, 70593 Stuttgart, Germany; 2 Institute for Social Medicine, Epidemiology and Health Economics, Charité – Universitätsmedizin Berlin, Luisenstraße 57, 10117 Berlin, Germany

## Abstract

**Background:**

Among food pantry users there is a high prevalence of both smoking and food insecurity, which may be related to one another. This study aims to evaluate the impact of a smoking cessation program carried out in food pantries on the smoking status and the food security status of food pantry users.

**Methods / design:**

Before starting the cluster randomised controlled trial, stakeholders will be engaged to adapt a behavioural group counselling program for smoking cessation to the needs of the food pantry users in a pre study. Food pantry users and workers as well as other experts, such as smoking cessation trainers, social workers, and psychologists, will be involved, using the world café technique and telephone interviews and a qualitative thematic analysis for data analysis to design the concept of the intervention program will be applied. In the second phase, the impact of the intervention on the smoking status and on food insecurity will be investigated by a cluster randomised controlled trial. A total of 416 food pantry users across 32 clusters (food pantries) in Berlin, Germany, should be recruited and randomly assigned to either the intervention group or the waiting list control group. The intervention will consist of a behavioural group counselling program for smoking cessation, specially tailored for food pantry users, as well as optional nicotine replacement therapy and the implementation of environmental smoking reduction measures in the food pantries. The primary outcomes 6 months after the treatment will be self-reported continuous smoking abstinence, validated by exhaled carbon monoxide (< 10 ppm of carbon monoxide), and increased food security level (the percentage of participants with an improved food security level).

**Discussion:**

This study will be the first long-term investigation into the effect of a smoking cessation program on smoking status and food insecurity. The results of this study will inform the implementation of smoking cessation programs in food pantries throughout Germany.

**Trial registration:**

Prospectively registered DRKS00020037. Registered 29 April 2020

## Background

Smoking is one of the major risk factors of mortality and morbidity in Germany [1] and accounts for around 125,000 premature deaths per year [2]. In addition to individual burden, smoking causes social costs of 79 billion euros per year [3].

Although the health risks of smoking are well known, around 28% of the population were reported to smoke in 2016 and 2017 in Germany [4]. Similar to other developed countries [5], smoking is not equally present among all demographics, but has become increasingly concentrated among low income population groups. For instance, from 1998 to 2014, the prevalence of smoking decreased in most income groups, whereas it increased to nearly 34% among the population group in the lowest income quartile [6]. Over the last decades, socio-economic differences in smoking behaviours have become one of the major contributors to existing socio-economic disparities in quality of life, morbidity and mortality [7, 8].

One population group at a particularly high risk of smoking is food pantry users. Food pantries are charitable organizations that distribute surplus food donated from food banks, retail stores, and manufacturers to those in need [9]. Although food pantry users are heterogeneous in terms of their educational background, age, and household size, one commonality is low income [10]. In previous studies in Germany, the prevalence of smoking was higher among participating food pantry users, at around 37% [11] and 47% [12], compared to others with low income. Similar or even higher smoking prevalence was found among food pantry users in other European countries [13, 14].

An additional concern is the observed high prevalence of food insecurity among food pantry users [11, 13–15]. Food insecurity exists “whenever the availability of nutritionally adequate and safe foods or the ability to acquire acceptable foods in socially acceptable ways is limited or uncertain” [16]. Food insecurity is related to dietary quality [17, 18] as well as the risk of several chronic diseases such as obesity, diabetes mellitus and depression [19, 20]. In a previous German study, around 70% of over 1000 food pantry users were reported to be food insecure, of which around 28% were moderately and 8% were severely food insecure [11]. In contrast, the Food and Agriculture Organization of the United Nations estimated that around 4% of the population was mildly or severely food insecure in Germany [21].

Thus food pantry users suffer disproportionately more from two considerable health risks, smoking and food insecurity, compared to the general population. Importantly, these health risks may be related, as recent studies among socio-economically disadvantaged individuals [22] and food pantry users [23] have found a significant relationship between smoking status and food insecurity, even when controlling for income or other socio-economic characteristics. For instance, a cross-sectional trend analysis demonstrated that in the USA from 1998 to 2011 the prevalence of food insecurity increased more among smokers compared to non-smokers [24]. One of the reasons might be that smokers with a low income use a disproportionately larger share of their income for cigarettes and other tobacco products than their wealthier counterparts [25]. This share of income is then not available for food. The struggle to afford both food and tobacco products might therefore increase the risk of food insecurity. In particular, among low-income individuals such as food pantry users, stopping smoking might lead to a significant relief of pantry users’ budgets and might, therefore, improve food security.

Several smoking cessation programs exist in Germany to assist people in stopping smoking [4]. As demonstrated by an international review, group behavioural therapy programs for smoking cessation are more effective than self-help [26]. However, only 12.5% of smoking cessation attempts are reinforced by evidence-based methods in Germany [4], which is considerably low compared to other countries [27]. Given that attempts to quit smoking without appropriate support have less chance of success (only 3–5% of self-quitters succeed in the long term [28]), a national health objective is to increase the use of evidence-based methods by implementing setting-based smoking cessation programs [29].

While smokers with low income do not differ in their usage of evidence-based smoking cessation methods from their wealthier counterparts [30], they succeed less often in smoking cessation in Germany [6, 31] and elsewhere [5]. Psychosocial factors contributing to these differences include lack of support, greater addiction to tobacco, and less motivation to quit [5]. However, as illustrated above, food pantry users might differ even from general low-income populations, and psychosocial factors related to smoking and smoking cessation among food pantry users are widely unknown. As a consequence, there is no smoking cessation program adapted to the needs of food pantry users.

Addressing both challenges, the low usage of smoking cessation programs and the lack of smoking cessation programs adapted to the needs of food pantry users, we will first adapt a smoking cessation group program according to the needs of food pantry users by stakeholder engagement and qualitative research. Then we will investigate the impact of this group program, when provided in parallel with nicotine replacement therapy and the implementation of environmental smoking reduction measures in the food pantries, on the smoking prevalence and food security among food pantry users in Berlin through a cluster randomised controlled trial.

## Objectives and hypotheses of the study

The main objective of the study is to investigate the impact of the smoking cessation measures on the smoking prevalence and on food security among food pantry users in Berlin.

The following primary hypotheses will be tested:

1. The percentage of individuals displaying continuous smoking abstinence 6 months after the treatment will be higher in the intervention condition compared to the control condition.
2. The percentage of participants with an improved food security level 6 months after the treatment will be higher in the intervention condition compared to the control condition.
3. The increased food security level will be mediated by continuous smoking abstinence.

Important secondary objectives include

- to assess the impact of the smoking cessation measures on other measures of smoking status (point prevalence of smoking abstinence, the mean number of cigarettes smoked per day) and of food insecurity (the mean number of affirmative responses on the FIES) as well as on the health-related quality of life scores and body-mass-index;
- to investigate whether possible intervention effects are moderated by demographics (age, gender, migration status, socioeconomic status), the nicotine dependence and time perspective.

## Methods / design

### Setting

Both the stakeholder engagement and the cluster randomised controlled trial will be conducted with the charitable organization the “Berliner Tafel” (“Berlin table”). At its start, the “Berliner Tafel” collected surplus foods from retailers and manufacturers and distributed these foods to social organizations such as women’s refuges, kids clubs, and homeless shelters. Since 2004, the “Berliner Tafel” has provided groceries to clients at 45 food pantries mainly located in religious establishments [32]. Those who fulfil the eligibility criteria defined by the “Berliner Tafel”, i.e. have an income at or below the level of the federal unemployment benefit II, are allowed to take groceries such as fresh fruits, vegetables, and bread for a small fee of one or two euros once a week or every second week [33]. Eligible individuals are allocated to one of the food pantries by officials of the “Berliner Tafel”, mostly according to residents’ postal code [33].

### Pre-study: stakeholder engagement to develop the intervention

Before starting the cluster randomised controlled trial, stakeholders will be engaged to develop a behavioural group counselling program for smoking cessation adapted to the needs of the food pantry users. The interventional group program is planned to be based on the existing evidence-based behavioural counselling smoking cessation program called “Rauchfrei” (“smokefree”) [34]. The needs of the food pantry users will be explored qualitatively in interviews and group discussions.

The original “Rauchfrei” program was developed by the Institute for Therapy Research in Munich and the German Federal Centre for Health Education (Bundeszentrale für gesundheitliche Aufklärung) and is the most widespread cognitive behavioural group program for smoking cessation in Germany [34]. The original program consists of 3 to 6 weekly meetings with other participants and the “Rauchfrei” trainer along with individual telephone-based counselling [35].

The methods of behavioural modification used consisted of cognitive strategies to change attitudes, motivational interviewing, strategies of goal orientation, and psychoeducation as well as the development of coping skills to deal with cravings and relapses. The program includes a fixed date to quit smoking in the middle of the program [35].

To identify the target group’s needs, the World Café technique [36] was chosen. One World Café with around 15 food pantry users, aged at least 18 years, will be conducted in a Berlin food pantry. Food pantry users will be recruited by leaflets, posters, and personal invitations from trained staff. The World Café will be conducted as follows: firstly, participants will be recruited through direct contact with a recruiter at their food pantry who will provide an informational brochure. They will be informed about the study’s aim and procedure, as well as about data protection regulation, verbally and with a written information sheet. If the pantry users are willing to participate they will be given a written consent form and, after signing it, will fill in a short questionnaire regarding demographics, smoking status, and including the quantity-frequency index [37]. The moderator will introduce participants to the World Café technique. Then groups of four or five participants will sit around four tables, together with a table host, and they will discuss freely predefined questions around the following four themes: 1. intentions and advantages of stopping smoking 2. barriers to stopping smoking 3. facilitators of stopping smoking 4. support from the food pantry to stop smoking. Each table host will have a different set of open-ended questions relating to one of these four main themes. After 20 min discussion participants will change the table and mix up to a new group. Participants will be encouraged to record or doodle their thoughts, comments, and questions on a white paper tablecloth. The table host will help the group to keep on topic and encouraged participation from all participants. At the end of the World Café, the hosts will present a summary of the discussions and all participants will discuss and reflect the findings.

Initially, an additionally second world café with around ten “Rauchfrei” trainers, food pantry managers, social workers, and psychologists was planned. Due to the ongoing COVID-19 pandemic, and the implementation of social distancing measures throughout Europe [38], semi-standardised phone interviews which each person alone will be conducted instead. The interview guide will contain the four main themes previously discussed by food pantry users during the World Café, as well as the experts’ concerns and knowledge in regards to food pantry users, smoking and smoking cessation. The formulation of the four main topics will be slightly adapted accommodate outsider perspectives on the situation of food pantry users. Food pantry users and managers participating in the formative study will receive a €20 voucher for an electronics shop or a clothing store.

Both the interviews and World Café discussions will be audio recorded digitally, transcribed, and will be analysed qualitatively by conducting thematic analysis with MAXQDA software (2018). In the next step themes and concepts relevant for smoking cessation in the context of food pantry users will be identified and then addressed through adaptations of the existing basic behavioural counselling smoking cessation program.

The results of the qualitative analysis will be discussed with experienced facilitators of smoking cessation programs in order to receive feedback. Based on the results of the qualitative analysis, relevant recent research, and the needs of the food pantry users, the behavioural group counselling intervention program for smoking cessation will then be designed.

### Study design

**Fig. 1:**
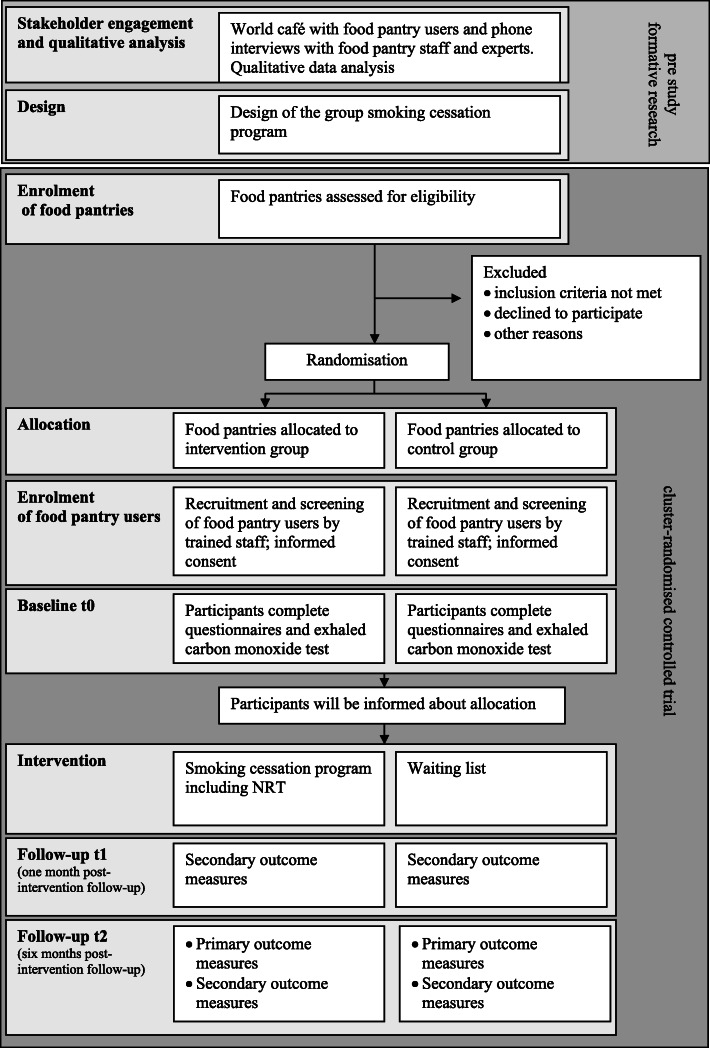
Flow chart of design and measurements

After the pre-study, a pragmatic two-arm cluster randomised controlled trial, targeting approximately 32 food pantries in Berlin, will be conducted. Cluster randomisation is used to minimise the risk of carryover effects from the intervention to the control group. Data among food pantry users will be assessed on the date of study inclusion (t0), 1 month after completing the smoking cessation program (t1), and 6 months after completing the smoking cessation program (t2) (see Fig. 1). The control group will receive the smoking cessation program after the intervention group has completed the program (waiting list control group).

### Study population

The study population will include users of any of the participating food pantries who reported smoking at least one cigarette or cigarillo per day. Participants must be at least 18 years old and able to sufficiently understand and speak German. Exclusion criteria includes non-smokers and individuals who smoke less than daily as well as individuals who do not use any of the participating food pantries. In addition, individuals who are 17 years of age or less, are unable to understand and speak German, or are illiterate, will also be excluded. Finally, individuals who only use e-cigarettes or tobacco heating systems will be excluded, since the impact of the original “Rauchfrei” program on this form of tobacco consumption is currently unknown.

### Procedures

#### Recruitment of the food pantries and randomisation

In consultation with the coordinator of the food pantries, all the “Berliner Tafel” food pantries will be invited to participate in the study by letters of invitation. Personal invitations will follow in case of no response.

Food pantries whose managers agree to participate via written consent will be randomly allocated to the intervention or control group by means of permuted block randomisation with randomly varying block sizes. The intended allocation ratio is 1:1. The randomisation lists will be computer generated. The team member conducting the randomization will be involved neither in recruitment of participants nor in data collection.

Given that participating food pantries will provide rooms for conducting the study for free, the pantries will be entered into a raffle for ten fuel vouchers valued at €50 each.

#### Recruitment of participants

Participants will be recruited after randomisation of the food pantries. First, users of participating food pantries will be informed by posters and leaflets. In addition, food pantry users will be personally invited by trained study staff.

#### Study procedure

While food pantry users are waiting in line to enter the room where the food is distributed, trained study staff will screen them for inclusion into the study using a checklist. Food pantry users willing to participate will be informed about the aim, the procedure of the study, and data protection measures, by a printed study information sheet. Individuals who are willing to participate will be asked to provide their name and address on a separate form and to complete the informed consent form as well as the questionnaire (t0). Exhaled carbon monoxide will also be measured. To secure blinding during the baseline data assessment, participants will be informed about the allocation of their food pantry on their next visit to the pantry (usually 1 week later) by the project coordinator.

One and 6 months after the intervention, follow up assessments will be conducted by trained staff. Again, participants will be assessed while waiting in line at the food pantry. Each food pantry will be visited twice. Participants who cannot be reached at the food pantry will receive the follow-up questionnaires by post.

Among all participants who complete the follow-up questionnaires, 50 €20 vouchers for either an electronics store or a clothing store will be raffled, irrespective of the participants’ smoking status.

#### Blinding

Due to the nature of the treatment conditions, it is impossible to blind the staff and participants throughout the study. However, the baseline assessment will be conducted while participants as well as staff are blinded.

### Intervention and control

#### Intervention

The intervention will consist of the smoking cessation program designed and adapted for food pantry users in the pre study as well as optional nicotine replacement therapy and the implementation of environmental smoking reduction measures in the food pantries.

The intervention will be provided by trained and certified smoking cessation trainers.

During the group sessions, potential environmental measures to make the food pantry less smoker friendly (e.g. defined smoking areas) will be identified and potentially implemented.

In addition to the group counselling, participants will be provided nicotine replacement therapy free of charge if requested. The dosage of the nicotine replacement will be individually tailored according to the Fagerström Test for Nicotine Dependence [39, 40].

#### Control

The control group consists of a waiting group and will be provided with the usual food pantry supplies and will receive the intervention when the last data collection period is finished (after t2).

### Outcome measures and evaluation

#### Measures

At t0, participants will be asked to complete a survey containing questions on demographics, developed by a previous study among food pantry users in Germany [41]; the Short Form 12 (SF-12) to measure the quality of life [42]; the quantity-frequency index [37]; the Fagerström Test for Nicotine Dependence (FTND) [39, 40]; the Food Insecurity Experience Scale (FIES) [43]; and the future, present-hedonistic and present-fatalistic subscales of the Zimbardo Time Perspective Inventory [44, 45]. At t1 and t2 continuous abstinence will be measured according to the “Russell standard” (“Have you smoked at all since [start date of last attempt to quit]?” “No, not a puff”; “1–5 cigarettes”; “More than 5 cigarettes?”) [46].

In addition, exhaled carbon monoxide will be measured at baseline as well as at t1 and t2. The time points for the measurements are shown in Table 1.

**Table 1:**
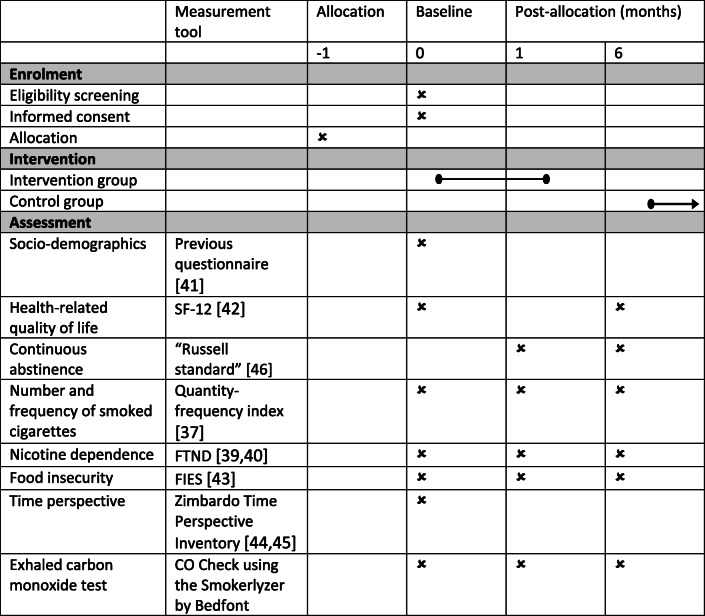
Time schedule of the cluster randomized trial

#### Outcomes

Primary outcomes are continuous smoking abstinence (yes or no; the percentage of participants with continuous smoking abstinence) and increased food security level (the percentage of participants with an improved food security level) 6 months after the treatment has finished. Smoking abstinence is defined as the self-reported continuous smoking cessation (≤ 5 cigarettes since the start date of their last attempt to quit) validated by the exhaled carbon monoxide test (< 10 ppm of carbon monoxide) [46]. Food insecurity is categorised into four groups: food security; mild food insecurity; moderate food insecurity; and severe food insecurity according to the Food and Agriculture Organization of the United Nations [21].

Secondary outcomes at one and 6 months post-intervention include the point prevalence of smoking abstinence, the mean number of cigarettes smoked per day, the mean number of affirmative responses on the FIES, and the health-related quality of life scores.

Time perspective will be investigated as moderator.

#### Process evaluation

Participation rate will be calculated. In addition, two focus groups will be conducted with members of the recruitment team, smoking cessation trainers, and study participants. The focus groups will aim to identify the advantages and disadvantages of the intervention barriers and facilitators to recruitment and compliance, and the overall therapeutic experience.

Finally, the number, type, and usage of all implemented environmental interventions will be documented monthly.

### Data management

At each food pantry, all participants will receive an identification number. Identifying information including the participant’s name, address and identification number will be kept separately and locked away from all other data and information. All data will be stored for 10 years. Only the research team will have access to trial data.

All original forms will be sent to the University of Hohenheim and will be entered electronically by trained staff. A subsample of original forms will be double entered by two independent scientists. Non-numeric data will be coded by using predefined standard terminology. Data integrity will be achieved by checking data ranges and valid values.

### Sample size

The required sample size was calculated using the function n4prop in the package CRTSize developed for the program R (version 3.3.2) [47].

In a previous study, the smoking status of users of eight food pantries in Berlin was assessed [11]. The number of participants per food pantry in Berlin was, on average, 56 ± 12 (ranging from 36 to 73) and the number of smokers was, on average, 26 ± 8 (ranging from 19 to 41). A systematic review of recruitment strategies of smoking cessation studies revealed that studies using “active strategies” (i.e. interpersonal contact with the research team or program provider) reached from 47% up to 84% of eligible individuals, with lower participation rates among low-educated populations [48]. Therefore, a participation rate of 50% and a sample size per cluster of *n* = 13 will be assumed.

Based on the results of previous studies [49], cessation rates of 10 and 3% are assumed among participants in the intervention group and the control group, respectively.

Assuming an intra-class correlation coefficient of 0.02, a 5% significance level, and 80% power, 32 clusters are needed to detect an effect (16 in the intervention group and 16 in the control group).

### Statistical analyses

The data will be analysed using the intention-to-treat principle, therefore including all participants irrespective of any dropouts after the baseline assessment. Missing values among the intention-to-treat population will be imputed using the baseline observation carried forward technique.

The mean, standard deviation, minimum and maximum will be given for continuous variables. Frequencies will be given for categorical variables.

To investigate differences between the intervention and the control group, independent t-tests for continuous variables and chi-square tests for categorical variables will be used. To test hypotheses 1 and 2, differences in the prolonged smoking abstinence and food insecurity improvement (the percentage of participants with an improved food security level) between the intervention and control group will be investigated using the chi-square test adjusted for intraclass correlation within clusters [50]. To account for the nesting of participants within the food pantry, mixed models with the logit link function will be used to predict cessation and food security improvement. To test hypothesis 3, a structural equation model will be used [51]. This identifies if the intervention condition predicts smoking cessation (path A), if smoking cessation predicts improvement in food security level (path B), if the intervention condition predicts the improvement in food security level (path C), and if the impact of the intervention condition on the improved food security level will be attenuated when controlling for smoking cessation.

Secondary outcomes will be analyzed accordingly.

### Ethical considerations

This study has been approved by the ethics committee of the University of Hohenheim (UHOH).

This research will be conducted in accordance with the Declaration of Helsinki (7th version, 2013) [52] and has been registered (DRKS00020037) [53].

The study is designed to minimise the risk to participants. Any unexpected events will immediately be reported to the ethics committee of the UHOH.

Potential participants will be given an information sheet in plain language as well as an informed consent form. Informed consent will be obtained from the participants prior to the enrolment into the study. The allocation of identification numbers will protect participants’ personal information (see above). Researchers will be reachable by email and phone over the entire study period. Participants will be allowed to withdraw from participation at any time without incurring a penalty.

## Discussion

This study will increase understanding into the impact of a smoking cessation program targeted to food pantry users on smoking status and food insecurity.

In Germany, more than 940 “Tafel” food banks and food pantries provide food to around 1.65 million economically disadvantaged people [54]. In other affluent countries, such as the USA [55] and the UK [56], millions of people also rely on the assistance of food pantries and food banks. The results of this study might assist in the development and implementation of smoking cessation interventions for food pantry users in comparable countries.

Moreover, this study will investigate, for the first time, the long-term effects of manipulating smoking status on food insecurity. One of the few longitudinal studies examining smoking status and food insecurity demonstrated that, among smokers who were food secure at baseline, becoming food insecure was independently associated with lower likelihood of smoking cessation at the 12 year follow-up. Moreover, among non-smokers who were food secure at baseline, becoming food insecure was significantly associated with a higher likelihood of starting smoking at the 12 year follow-up, even when controlling for demographic variables including poverty [57].

The majority of people who started smoking after baseline were former smokers, indicating that food insecurity may increase the risk of relapse. Thus, food insecurity may not only be a consequence of smoking but also a risk factor for smoking relapse due to stress, hunger, or other factors related to food insecurity which smoking may alleviate [57]. The presented study aims to mitigate the impact of food insecurity on smoking relapse by involving food insecure participants in the development of a smoking cessation program. In the group sessions, known risk factors for relapse, such as food insecurity, will be addressed and participants will acquire strategies to cope with stress situations.

The results of this study will contribute to an enhanced understanding of the interplay between smoking status and food insecurity, and inform the practices of professionals who provide interventions to reduce the health risks in this population group, including social workers, psychologists and health professionals.

### Strengths and limitations

One of the most important strengths of this study is the stakeholder engagement within the food pantry setting. Over the last few years, food pantries in Germany have broadened their mandate from solely distributing food to establishing central meeting points where clients are able to drink coffee, eat snacks, and meet peers while waiting to receive their food [58]. As revealed by a previous study, the majority of users regularly visit a food pantry at least four times per month for more than 1 year [11]. This makes food pantries an ideal setting for interventions aimed at a population group that is otherwise hard to reach. By providing the smoking cessation program in the same place as the food pantry, demands and barriers will be minimised for the participants.

This study is innovative as it adapts an already existing well-established smoking cessation program to the specific needs of food pantry users. Furthermore, this study is needed to address health inequalities within an underserved population group.

The “Tafel” food pantries are strongly connected and collaborate under the federal association called “Tafel Deutschland” [54]. If the intervention is effective, this network will enable the expansion of the intervention throughout the country.

Despite the thoroughly planned design, this study is not without limitations. For instance, there is the possibility of bias due to the unblinded design of the study after the baseline assessment. Participants in the control group might be disappointed upon the realisation that they have to wait for the intervention, potentially leading to attrition bias.

In addition, designs using waiting list controls have been criticised as they may overestimate intervention effects [59]. Participants, in particular those who are ready to change, may stop or delay their efforts to change during the waiting period [60]. Using an active control could prevent potential nocebo effects but an equivalent smoking cessation program appropriate for food pantry users, or even low-income people in general, was not available*.* In addition to the lack of an appropriate program for comparison, the program implementation is very complex and expensive which made the researchers decide on a waiting list control group.

Furthermore, it will not be possible to assess the relative contribution of each study component (the group counselling, the nicotine replacement therapy and the environmental measures) to the intervention’s impact on smoking behaviours and food insecurity.

The intervention will be free of charge for participants. It could be argued that free interventions are unlikely to be offered outside of research settings, and that self-payment of an intervention might increase its success (compared to the dissonance theory [61]). However, Curry et al. showed that cessation program utilisation was highest when fully covered by insurance, although full coverage was also associated with a slightly lower abstinence rate than the other coverage models. Therefore full coverage models had the greatest impact on smoking prevalence [62].

One of the challenges might be recruitment. A large number of clusters, and therefore participating food pantries, are needed to detect an effect. The focus of food pantries lies on the distribution of food and nearly all “Tafel” food banks and food pantries are driven by volunteers [58]. Although the coordinator of all food pantries will be involved in the recruitment and written invitations will be followed by personal invitations in the case of no response, the recruitment of 32 food pantries remains ambitious, in particular in times of the ongoing COVID-19 pandemic [38].

## Data Availability

Not applicable.

## Abbreviations

CO: Carbon monoxide
FIES: Food Insecurity Experience Scale
FTND: The Fagerström Test for Nicotine Dependence
SF-12: 12 item Short Form Health Survey
UHOH: University of Hohenheim
